# Diabetes Mellitus and Neurovascular Pathology: A Comprehensive Review of Retinal and Brain Lesions

**DOI:** 10.7759/cureus.70611

**Published:** 2024-10-01

**Authors:** Gayathri Donthula, Sachin Daigavane

**Affiliations:** 1 Ophthalmology, Jawaharlal Nehru Medical College, Datta Meghe Institute of Higher Education & Research, Wardha, IND

**Keywords:** brain lesions, diabetes mellitus, diabetic retinopathy, hyperglycemia, neurovascular damage, retinal lesions

## Abstract

Diabetes mellitus (DM) is a chronic metabolic disorder marked by persistent hyperglycemia, which significantly impacts vascular health. This review comprehensively analyzes the neurovascular complications associated with DM, focusing on retinal and brain lesions. Diabetes is categorized into type 1 DM, type 2 DM, and gestational DM, each presenting unique challenges and risks. The condition accelerates vascular damage through mechanisms such as endothelial dysfunction, inflammation, and oxidative stress, leading to severe microvascular complications. Diabetic retinopathy is a primary concern, with its progression from non-proliferative to proliferative stages potentially resulting in vision loss. Concurrently, diabetes contributes to neurovascular damage in the brain, increasing the risk of cognitive decline and cerebrovascular events. This review examines the pathophysiological mechanisms underlying these complications, evaluates current diagnostic and management strategies, and highlights recent advancements in imaging technologies and therapeutic approaches. Integrating these insights is crucial for improving early detection, treatment, and management of diabetes-related neurovascular issues. Future research should focus on innovative preventive measures and therapeutic interventions to mitigate the long-term impact of diabetes on vascular health. By enhancing our understanding of these complex interactions, this review aims to contribute to better clinical practices and improved patient outcomes in diabetes care.

## Introduction and background

Diabetes mellitus (DM) is a chronic metabolic disorder characterized by persistent hyperglycemia, which results from defects in insulin secretion, insulin action, or both [1]. The condition leads to impaired glucose metabolism and, if left untreated or poorly managed, can have severe long-term health consequences. DM is categorized into several types: type 1 DM, type 2 DM, and gestational DM (GDM) [2]. Type 1 diabetes is an autoimmune disorder where the body’s immune system attacks and destroys pancreatic beta cells, leading to absolute insulin deficiency. It typically presents in childhood or adolescence and necessitates lifelong insulin therapy [3]. Type 2 diabetes (T2D), the most prevalent form, is characterized by insulin resistance and a relative insulin deficiency. It commonly develops in adults, though rising obesity rates have led to an increase in cases among children and adolescents. Management often involves lifestyle changes and oral medications, with insulin therapy becoming necessary in advanced stages [4]. Gestational diabetes occurs during pregnancy and is diagnosed when a previously nondiabetic woman exhibits high blood glucose levels. While GDM generally resolves after delivery, women with a history of GDM are at an elevated risk of developing T2D later in life [4].

The prevalence of DM has reached alarming levels globally. According to the International Diabetes Federation, over 460 million adults are currently living with diabetes, and this number is expected to rise significantly in the coming decades [5]. This growing prevalence places a substantial burden on healthcare systems and contributes to increased morbidity and mortality rates worldwide. Consequently, understanding the broader implications of diabetes on health, including its effects on vascular systems, becomes crucial [5]. One of the critical areas of concern in DM is its impact on the neurovascular system, encompassing both retinal and brain vessels. Diabetes significantly affects vascular health due to chronic hyperglycemia, which contributes to endothelial dysfunction, inflammation, and oxidative stress [6]. These factors collectively accelerate vascular damage and are implicated in the development of neurovascular complications. Diabetic retinopathy (DR), a common microvascular complication, is a leading cause of vision loss resulting from damage to the retinal blood vessels [7]. DR can progress from mild non-proliferative stages to severe proliferative forms, potentially leading to blindness if not properly managed. Similarly, diabetes is associated with an increased risk of cerebrovascular disease, including stroke and cognitive decline. Microvascular damage in the brain can lead to significant neurological impairment, making it a major concern in diabetes management [7].

Understanding these neurovascular complications is critical for improving patient care. Both retinal and brain lesions significantly impact the quality of life and overall health outcomes for individuals with diabetes. Early detection and effective management of these complications are essential for mitigating long-term health risks and enhancing patient well-being [6]. This review aims to comprehensively analyze the neurovascular pathology associated with DM, focusing on retinal and brain lesions. By exploring the underlying mechanisms, diagnostic methods, and treatment strategies, the review seeks to advance understanding and inform clinical practice. Additionally, it will highlight current research trends and future directions, underscoring the importance of integrated approaches in managing diabetes and its neurovascular risks [8].

## Review

Pathophysiology of DM

**Figure 1:**
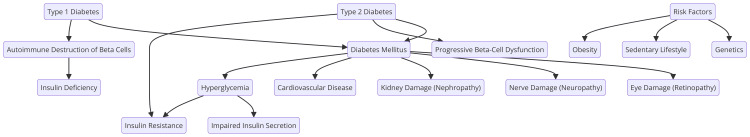
Pathophysiology of DM DM, diabetes mellitus Image credit: Gayathri Donthula

DM, particularly T2D, is marked by chronic hyperglycemia due to insulin resistance and impaired insulin secretion. This review highlights the key mechanisms underlying the pathophysiology of diabetes, focusing on hyperglycemia and insulin resistance, inflammation and oxidative stress, and endothelial dysfunction [9]. Hyperglycemia in diabetes primarily results from insulin resistance, where tissues such as muscle and adipose fail to respond adequately to insulin. This resistance is often associated with obesity and is further exacerbated by factors such as inflammation and oxidative stress. Persistent hyperglycemia leads to the formation of advanced glycation end-products, which further disrupt insulin signaling pathways [10]. The metabolic consequences include increased hepatic glucose production and decreased glucose uptake by peripheral tissues, perpetuating elevated blood glucose levels. Research indicates that inflammatory cytokines, such as interleukin-6 and tumor necrosis factor-alpha, play a crucial role in developing insulin resistance by fostering a state of chronic low-grade inflammation, characteristic of T2D [11]. Inflammation and oxidative stress are interrelated processes that significantly contribute to the complications associated with diabetes. Hyperglycemia induces oxidative stress by overproducing reactive oxygen species, damaging cellular structures, and triggering inflammatory responses. This oxidative stress is linked to the activation of inflammatory pathways, leading to the release of cytokines that exacerbate vascular damage [12]. For example, the chronic elevation of pro-inflammatory cytokines can lead to endothelial dysfunction, a precursor to vascular complications such as atherosclerosis and diabetic nephropathy. Additionally, oxidative stress not only contributes to diabetes progression but also plays a pivotal role in developing its complications, including cardiovascular diseases. The interplay between oxidative stress and inflammation creates a vicious cycle that worsens tissue damage and insulin resistance [13]. Endothelial dysfunction is a significant consequence of diabetes that impacts vascular health. It is characterized by a diminished ability of the endothelium to regulate blood flow and maintain vascular homeostasis. In diabetes, elevated levels of glucose and lipids, combined with increased oxidative stress, impair endothelial nitric oxide synthase, essential for producing nitric oxide (NO) [14]. NO is a critical vasodilator that helps regulate vascular tone and prevent platelet aggregation. The reduction in NO bioavailability contributes to increased vascular resistance, promoting hypertension and elevating the risk of cardiovascular events. Furthermore, endothelial dysfunction is associated with increased permeability and inflammation, which facilitates the development of atherosclerosis and other vascular complications [15]. The pathophysiology of DM is complex, involving hyperglycemia, insulin resistance, inflammation, oxidative stress, and endothelial dysfunction. Understanding these mechanisms is essential for developing effective therapeutic strategies to manage diabetes and its associated complications. Ongoing research into the interactions among these factors will aid in identifying novel intervention targets and improving patient outcomes [16]. The pathophysiology of DM is illustrated in Figure 1.

Retinal lesions in DM

DM, especially T2D, can result in a range of retinal lesions collectively referred to as DR. DR is a major cause of vision loss among working-age adults and is categorized into two primary stages: non-proliferative DR (NPDR) and proliferative DR (PDR) [17]. NPDR represents the initial stage of DR, characterized by microaneurysms (small balloon-like bulges in the retinal blood vessels), hemorrhages (small bleeding areas from damaged vessels), exudates (fatty deposits from leaking blood vessels), and macular edema (swelling of the macula due to fluid leakage, which is the most common cause of vision loss in DR). As NPDR advances, more blood vessels become obstructed, leading to oxygen deprivation (ischemia) in the retina. In response, the retina releases growth factors that stimulate the formation of new blood vessels [18]. PDR is the advanced stage of DR, marked by the development of new, abnormal blood vessels (neovascularization) on the retina’s surface or the optic nerve, scar tissue formation (which can result in retinal detachment and severe vision loss), and vitreous hemorrhage (bleeding into the clear, gel-like substance filling the eye) [19]. Various imaging techniques are employed to diagnose and monitor DR, including fundus photography (which captures retinal images to detect lesions such as microaneurysms, hemorrhages, and exudates), optical coherence tomography (OCT) (which uses light waves to obtain cross-sectional images of the retina, allowing for the detection of macular edema and other changes), and fluorescein angiography (which involves injecting a dye into the bloodstream to highlight areas of retinal blood vessel leakage or closure) [20]. The primary objectives in managing DR are to maintain tight glycemic control (as strict regulation of blood sugar levels can significantly reduce the risk and slow the progression of DR) and to address vision-threatening DR (using laser therapy to treat macular edema and prevent vision loss in advanced NPDR and PDR and anti-vascular endothelial growth factor injections to reduce abnormal blood vessel growth and leakage in PDR and diabetic macular edema). Regular eye examinations are essential for early detection and timely treatment of DR, which can reduce the risk of severe vision loss by up to 90% when managed appropriately [21].

Brain lesions in DM

DM has a profound impact on brain health, leading to various neurovascular complications, including diabetic encephalopathy, neurovascular damage, and cognitive impairment. Diabetic encephalopathy is marked by a range of pathological changes in the brain, primarily due to the effects of hyperglycemia on cerebral microvascular integrity. These changes include cerebral microangiopathy, which involves damage to small blood vessels, resulting in reduced blood flow and nutrient delivery to brain tissues [22]. The resulting ischemia can cause neuronal injury and death, contributing to cognitive decline. Additionally, diabetes is associated with an increased burden of white matter lesions (WMLs), which appear as hyperintensities on MRI scans and are indicative of small vessel disease. These lesions are linked to cognitive impairment, particularly affecting processing speed and executive function. Cerebral microbleeds, which are small, chronic hemorrhages resulting from the fragility of the microvasculature in diabetic patients, also contribute to neurodegeneration and cognitive deficits [23]. Diabetes is associated with an elevated risk of cognitive impairment and dementia. Studies have demonstrated that individuals with diabetes experience significant declines in cognitive domains such as memory, attention, and executive function. The risk of developing vascular dementia is notably higher in diabetic patients, with relative risks estimated at 2.5 for vascular dementia and 1.5 for Alzheimer’s disease (AD) compared to nondiabetic individuals [24]. Small vessel disease, a common consequence of diabetes, leads to various neurovascular lesions, including lacunar infarcts - small, deep brain infarcts often linked to chronic hypertension and diabetes. These infarcts contribute to cognitive decline and an increased risk of stroke. The presence of WMLs and cerebral microbleeds reflects the extent of neurovascular damage in diabetic patients. It disrupts normal brain function, correlating with cognitive decline, especially in older adults with diabetes [25]. MRI is the primary imaging modality used to assess brain lesions in diabetic patients. It can detect WMLs, identified as hyperintensities on T2-weighted images, correlating with cognitive deficits. MRI can also identify microbleeds, which are crucial for understanding the extent of vascular damage in the brain [26]. Effective management of blood glucose levels is essential in preventing or mitigating brain lesions associated with diabetes. Maintaining glycemic control can help reduce the risk of microvascular complications and subsequent cognitive decline. Given the strong connection between cardiovascular health and brain function, strategies aimed at reducing cardiovascular risk factors - such as hypertension, hyperlipidemia, and smoking - are vital. Lifestyle modifications, including a healthy diet and regular physical activity, can significantly improve metabolic and cognitive outcomes in diabetic patients [27].

Comparative analysis of retinal and brain lesions

Retinal and brain lesions share several common risk factors, particularly in conditions such as AD and DM. Age is a significant risk factor for both retinal and brain pathologies, with neurodegenerative changes becoming more prevalent in older populations [28]. Diabetes is also closely linked to both DR and cognitive decline, as studies suggest that retinal vascular changes in diabetes can predict the risk of cerebrovascular diseases and cognitive impairment. Genetic factors further contribute to these conditions; for instance, the ApoE ε4 allele has been associated with both retinal and brain changes in AD, indicating a potential genetic overlap in their pathophysiology [29]. The mechanisms connecting retinal and brain pathology are complex. Neurovascular dysfunction is a crucial factor affecting both the retina and brain, leading to reduced blood flow and subsequent neuronal damage. This dysfunction is particularly evident in conditions like AD and diabetes, where both retinal and cerebral blood flow are compromised [30]. Chronic inflammation and oxidative stress are common pathways contributing to neurodegeneration in the retina and brain. These processes can lead to the accumulation of amyloid plaques and tau tangles in AD, also observed in retinal tissues. Studies have shown that retinal amyloid-beta (Aβ) levels correlate with brain Aβ levels, suggesting that retinal imaging could serve as a noninvasive biomarker for brain pathology in AD [31]. The implications of retinal findings for brain health are significant, especially for early detection and intervention. Retinal imaging techniques, such as OCT, have become valuable tools for assessing brain health. Changes in retinal structure, such as thinning of the retinal nerve fiber layer (RNFL) and alterations in macular thickness, have been correlated with brain atrophy and cognitive decline. For example, reduced RNFL thickness has been associated with cortical atrophy in Alzheimer’s patients, indicating that retinal changes may serve as early indicators of neurodegeneration [32]. Furthermore, retinal findings can act as predictive markers for cognitive decline. Research has shown that specific retinal changes can signal the presence of underlying brain pathology, allowing for earlier intervention and management strategies. This noninvasive approach to assessing retinal pathology can provide critical insights into the neurodegenerative processes occurring in the brain, making it a potential biomarker for diseases like AD and other forms of dementia. The correlation between retinal and brain pathology highlights the importance of integrating ocular assessments into evaluating neurodegenerative diseases, facilitating early diagnosis, and potentially improving patient outcomes [33].

Current research and future directions

Recent innovations in imaging technologies have markedly improved the visualization and diagnosis of diabetic complications, particularly in the retina and brain. In retinal imaging, techniques such as OCT and fundus autofluorescence are being refined to detect early structural and functional changes before the onset of DR [20]. These advancements enable the identification of neurovascular impairments and structural alterations in the retina, allowing for timely intervention. Early detection is crucial as it can lead to better management strategies that may prevent or slow the progression of DR [34]. In brain imaging, advanced neuroimaging techniques, including functional MRI and diffusion tensor imaging, assess cerebral blood flow and white matter integrity in diabetic patients. These modalities help elucidate the relationship between diabetes and cognitive decline by identifying changes in brain connectivity and microstructural integrity. The ability to visualize these changes in real-time enhances our understanding of how diabetes impacts the brain and opens avenues for targeted therapeutic interventions [35]. The treatment landscape for diabetes is evolving with the introduction of novel pharmacological therapies to improve glycemic control and reduce complications. Recent studies highlight the efficacy of sodium-glucose co-transporter 2 inhibitors and glucagon-like peptide-1 receptor agonists [36]. These agents improve glycemic control and offer cardiovascular and renal protection, addressing multiple aspects of diabetes management. By targeting both metabolic and vascular health, these therapies represent a paradigm shift in diabetes treatment, with the potential to mitigate the risk of neurovascular complications [36]. Moreover, the application of nanotechnology in diabetes treatment is gaining momentum. Developments in nanocarrier systems aim to enhance drug delivery and bioavailability, potentially leading to improved management of diabetes and its complications. As research progresses, more personalized and effective treatment options tailored to the unique needs of individuals with diabetes may emerge [37]. Preventive strategies are crucial in managing diabetes and its associated complications. Emphasizing lifestyle changes, such as diet and exercise, is essential for diabetes management. Studies indicate that weight management and physical activity can significantly reduce the risk of complications such as DR and cognitive decline. By promoting a healthy lifestyle, healthcare providers can empower patients to take charge of their health and potentially delay or prevent the onset of serious complications [38]. In addition to lifestyle modifications, implementing screening programs for early detection of diabetic complications is critical. Enhanced imaging techniques and biomarkers are being explored to identify at-risk individuals before clinical symptoms manifest. Early intervention strategies can lead to better diabetes management and its neurovascular complications. By focusing on prevention and early detection, healthcare systems can improve patient outcomes and reduce the overall burden of diabetes-related diseases [39]. An overview of current research and future directions in the neurovascular pathology of DM is presented in Table 1.

**Table 1:** Overview of current research and future directions in neurovascular pathology of DM DM, diabetes mellitus; DR, diabetic retinopathy; OCT, optical coherence tomography; PDR, proliferative diabetic retinopathy; VEGF, vascular endothelial growth factor; WML, white matter lesion

Focus area	Current research	Future directions
Neurovascular pathology [24]	Studies on the relationship between cognitive decline and diabetic encephalopathy. Exploration of the role of systemic inflammation in linking DR and brain lesions	Longitudinal studies to understand the progression of neurovascular pathology in diabetes over time. Investigating new biomarkers for early detection of diabetic encephalopathy
Advances in imaging technologies [40]	Development of high-resolution OCT for retinal imaging. Advanced MRI techniques for detecting brain microbleeds and WMLs in diabetic patients	Integration of AI for early detection of neurovascular lesions. Development of noninvasive imaging tools for more sensitive and specific detection of microvascular damage
Emerging therapies [41]	Anti-VEGF therapy for PDR. Pharmacological interventions targeting oxidative stress and endothelial dysfunction in diabetic encephalopathy	Exploration of gene therapy and personalized medicine approaches to treat neurovascular damage. Clinical trials on combination therapies targeting retinal and brain lesions in diabetes
Preventive strategies [42]	Ongoing research into lifestyle interventions like diet and exercise for reducing the risk of neurovascular complications in diabetic patients	Developing targeted prevention programs incorporating genetic and biomarker screening to predict individual risk. Enhanced public health strategies to promote early detection and prevention of diabetes-related neurovascular complications
Integrated management approaches [43]	Research combining blood sugar management with cardiovascular risk reduction strategies to mitigate neurovascular complications	Designing comprehensive care models that integrate retinal and brain health monitoring for diabetic patients
Technological innovations [44]	Development of smart contact lenses for continuous glucose monitoring and early detection of DR	Advancement in wearable technologies for real-time monitoring of neurovascular health in diabetic patients

## Conclusions

DM represents a significant global health challenge with profound implications for vascular health, particularly concerning neurovascular pathology. The chronic hyperglycemia associated with diabetes contributes to extensive vascular damage, manifesting prominently in both retinal and brain lesions. DR, with its potential to progress from mild to severe forms, poses a major risk to vision and quality of life. At the same time, neurovascular damage in the brain can lead to serious neurological impairments and an increased risk of cerebrovascular events. Effective management of these complications requires a comprehensive understanding of their pathophysiology, vigilant monitoring, and timely intervention. This review has aimed to illuminate the intricate relationship between diabetes and neurovascular damage, highlighting the need for ongoing research and the integration of advanced diagnostic and therapeutic strategies. By enhancing our grasp of these complex interactions, we can better address the multifaceted challenges posed by diabetes, ultimately improving patient outcomes and reducing the burden of this chronic condition. Future research should continue to explore innovative approaches to prevention, early detection, and treatment, ensuring that individuals with diabetes receive the best possible care and support.
